# Combination of Immune Checkpoint Inhibitors and Anti-Angiogenic Agents in Brain Metastases From Non-Small Cell Lung Cancer

**DOI:** 10.3389/fonc.2021.670313

**Published:** 2021-05-04

**Authors:** Likui Fang, Wuchen Zhao, Bo Ye, Da Chen

**Affiliations:** Department of Thoracic Surgery, Affiliated Hangzhou Chest Hospital, Zhejiang University School of Medicine, Hangzhou, China

**Keywords:** non-small cell lung cancer, brain metastases, immune checkpoint inhibitors, anti-angiogenesis, combination therapy

## Abstract

Brain metastases remain a critical issue in the management of non-small cell lung cancer (NSCLC) because of the high frequency and poor prognosis, with survival rates often measured in just months. The local treatment approach remains the current standard of care, but management of multiple asymptomatic brain metastases always involves systemic therapy. Given that anti-angiogenic agents and immune checkpoint inhibitors (ICIs) both target the tumor microenvironment (TME), this combination therapy has become a promising strategy in clinical practice. Increasing number of preclinical and clinical studies have shown remarkable anti-tumor activity of the combination therapy, but the efficacy in brain metastases is unclear due to the strict selection criteria adopted in most clinical trials. This review briefly summarizes the potential synergistic anti-tumor effect and clinical development of the combination of anti-angiogenic agents and ICIs in NSCLC brain metastases, and discusses the existing challenges and problems.

## Introduction

Lung cancer is one of the most common malignant tumors and the leading cause of cancer-related mortality worldwide (1, 2). Non-small cell lung cancer (NSCLC) is the most frequent subtype of lung cancer and approximately 57% patients with NSCLC are in advanced stage including 20% presenting with brain metastases at the time of diagnosis (3). Brain metastases are also the common pattern of distant relapse after initial treatment (4, 5). Brain metastases are associated with poor prognosis and portend limited effective treatment options (6).

Current treatment strategies include local and systematic management. For the patients with symptomatic and immediately life-threatening brain metastases, surgical resection and radiotherapy are the major therapeutic approaches because of their relatively effective local control (7–9). However, surgery is typically reserved for intracranial hemorrhage, large lesions, and solitary brain metastases (10). Similarly, the use of stereotactic radiosurgery is limited by the number of metastatic lesions and is not suitable for the tumors which are larger than 4cm or located in critical structures (11, 12). Whole brain radiotherapy is still the main method for the patients with multiple brain metastases or when stereotactic radiosurgery is not feasible (13). Although local treatment has an irreplaceable status in brain metastases currently, its toxic effects should warrant enough attention, such as cognitive decline and symptomatic radiation necrosis (7, 14, 15). Moreover, local treatment could delay the initiation of systemic treatment, which would lead to the progression of primary tumors and compromise long-term outcomes.

Considering the limitations of local treatment, systematic therapy for NSCLC brain metastases has been explored due to its simultaneous treatment for both intracerebral and extracerebral diseases. Chemotherapy is not so often an effective approach for metastatic brain lesions, whereas tyrosine kinase inhibitors (TKIs) therapy such as Osimertinib in oncogene driven disease has shown a good activity also on brain metastases (16–18). In the era of immunotherapy, there is increasing evidence supporting the use of immune checkpoint inhibitors (ICIs) in the treatment of NSCLC brain metastases when no targetable driver mutation has been identified (19). Despite the encouraging data, only few patients respond to immunotherapy and additional combination treatment strategies are in urgent need. Given that both anti-angiogenesis and immune checkpoint blockade focus on targeting the tumor microenvironment (TME), the combination of ICIs and anti-angiogenic agents has become an attractive strategy. This review summarizes the potential synergistic anti-tumor effect and clinical development of this combination therapy strategy in NSCLC brain metastases.

## Potential Mechanisms

Tumorigenesis involves a succession of genetic alterations which have been classified into eight distinctive and complementary biologic capabilities, including sustaining proliferative signaling, evading growth suppressors, deregulating cellular energetics, enabling replicative immortality, resisting cell death, inducing angiogenesis, avoiding immune destruction and activating invasion and metastasis (20). Therefore, angiogenesis and immune escape are two critical processes of tumorigenesis. Moreover, TME is widely accepted as an important regulator of cancer formation and progression. The tumor vasculature is a key component of the microenvironment that can be targeted through the use of anti-angiogenic agents. Blood vascular and lymphatic endothelial cells have important roles in regulating the microenvironment and modulating the immune response. Improving access to the tumor through vascular normalization with anti-angiogenic agents may prove an effective combination strategy with immunotherapy approaches, and this combination therapy could have synergistic effects on TME to inhibit tumorigenesis. However, even though TME is a potentially rich source of therapeutic targets, our knowledge of the brain TME lacks comprehensive and integrative analysis.

The brain has long been regarded as immune privileged organ because blood brain barrier (BBB) and blood cerebrospinal fluid barrier (BCB) limit the entry of immune cells from the periphery. However, the immune privileged status of brain has been recently challenged by the discovery of lymphatic vessels that connect the central nervous system (CNS) with the periphery and are able to carry both fluid and immune cells (21, 22). This discovery leads to a reassessment of long-held assumptions in neuroimmunology and sheds new light on the application of immunotherapy in brain metastases. Several in-depth studies of immune microenvironmental landscape within CNS have revealed disease-specific enrichment of immune cells, including tissue-resident microglia, infiltrating monocyte-derived macrophages, neutrophils, and T cells (23, 24). Principal-component analysis has confirmed that monocyte-derived macrophages, neutrophils, and CD4+ and CD8+ T cells are the major immune cell determinants of the TME landscape of lung cancer brain metastases (24). In addition, brain metastases can disrupt the integrity of the BBB and BCB and recruit different immune cells from the myeloid and lymphoid lineage to the CNS (25). Angiogenesis is one of the specific hallmarks of NSCLC brain metastases and pivotal for the progression of metastasizing lesions, which have been proven by the observations of human autopsy specimens (26, 27). In addition, the tumor vasculature has important immunomodulatory roles including preventing the immune rejection of tumors (28). There have been several clinical studies suggesting that inclusion of anti-angiogenic therapies should be evaluated in selected patients with asymptomatic NSCLC brain metastases (29, 30). These findings provide theoretical supports for the use of ICIs and anti-angiogenic agents in NSCLC brain metastases. The development of this combination strategy is based on the understandings of the interaction between these two therapeutic interventions and their effects on the TME.

### Anti-Angiogenic Agents Promote Anti-Tumor Immune Response

**Figure 1:**
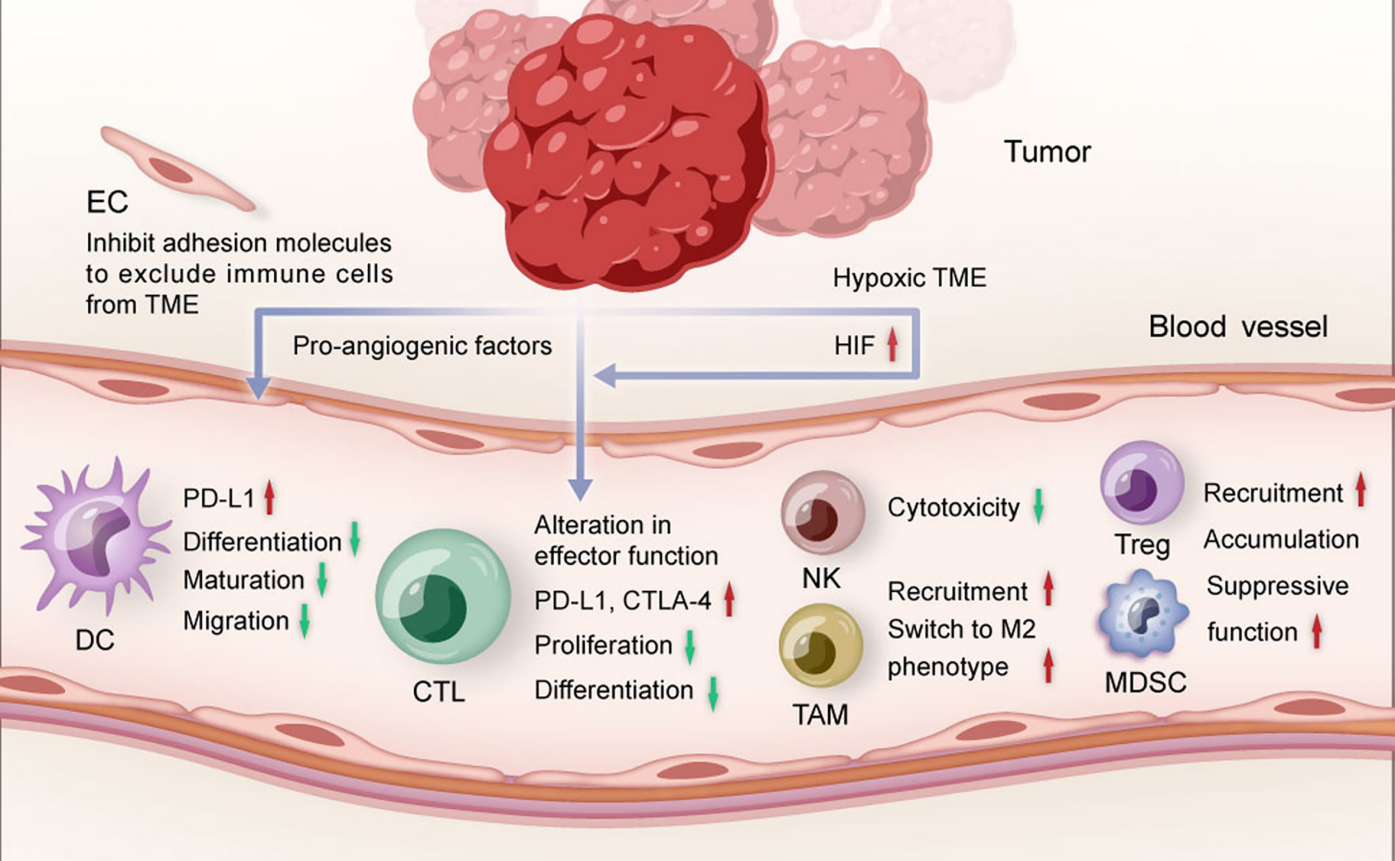
The role of tumor angiogenesis in TME. Pro-angiogenic factors and hypoxia restrict the maturation and migration of dendritic cells, reduce the proliferation and differentiation of effector CTLs, and promote the recruitment of suppressive immune cells. TME, tumor microenvironment; DC, dendritic cell; CTL, cytotoxic T lymphocytes; NK, natural killer cell; TAM, tumor-associated macrophage; Treg, regulatory T cell; MDSC, myeloid-derived suppressor cell; EC, endothelial cell; HIF, hypoxia-inducible factor.

Angiogenesis involves many signaling pathways, such as vascular endothelial growth factor (VEGF)-VEGF receptor (VEGFR), platelet derived growth factor (PDGF)-PDGF receptor (PDGFR) and fibroblast growth factor (FGF)-FGF receptor (FGFR). These signaling pathways influence multiple steps of the cancer immune response (31, 32) (**Figure 1**). VEGF is one of the most studied factors triggering angiogenesis. In the circulation, the level of VEGF was found to be inversely correlated with the level of mature dendritic cell (DC) which is the main antigen-presenting cell (33). VEGF-VEGFR signaling pathway could inhibit the transcriptional activation of nuclear factor-κB to affect the differentiation and maturation of DCs (34, 35). Moreover, VEGF could also inhibit the antigen-presentation function of DCs by upregulating programmed cell death protein ligand-1 (PD-L1) expression on DCs (36). As a result, cancer antigens fail to be presented to T cells, leading to silence of cytotoxic T lymphocytes. In addition, PDGF could also restrain DC maturation (31). Anti-angiogenic agents could increase the level of mature DCs and enhance the uptake of antigen presentation, resulting in the promotion of anti-tumor immune response (37, 38).

T cell infiltration is widely accepted as a key component of adaptive cancer immune response. VEGF could inhibit the differentiation of CD8+ and CD4+ T cells from hematopoietic progenitor cells and lead to the occurrence of T-cell deficiency (39). Moreover, activation of VEGF-VEGFR signaling pathway on CD8+T cells could induce T cell exhaustion and reduce T cell cytotoxicity by increasing the expression of programmed cell death protein 1 (PD-1) and cytotoxic T-lymphocyte-associated protein 4 (CTLA-4) (40). Similarly, natural killer cells (NK) cytotoxicity could be impaired by VEGF-VEGFR signaling pathway (41). Overexpressed VEGF could also inhibit the recruitment of type 1 helper T cells (Th) at tumor site but enhance the recruitment and proliferation of immunosuppressive cells including regulatory T cells (Treg) and myeloid-derived suppressor cells (MDSC) to promote the formation of immunosuppressive microenvironment (31).

The steps of immune cells infiltrating into the TME include entering the tumor vessels, attaching to the endothelial cells and finally migrate to the TME through the vascular wall (42). Angiogenic molecules are capable to regulate the expression of different adhesion molecules such as intercellular adhesion molecule-1 (ICAM-1) and vascular cell adhesion molecule 1 (VCAM1) to inhibit the transfer of immune cells to TME (28). Moreover, tumor endothelial cells could not only form a specific selective barrier to inhibit the penetration of certain immune cells (31), but also modulate the activity and variability of immune cells to regulate immunosuppression (32).

Abnormal tumor vasculature could aggravate the hypoxia in TME, leading to immune suppression through multiple mechanisms including recruitment of MDSCs, accumulation of Tregs (43) and activation of hypoxia-inducible factor (HIF) which is a critical factor of regulating angiogenesis and immune response (44). HIF could participate in innate and adaptive immunity. For example, HIF could promote recruitment of monocytes and M2 tumor-associated macrophages (TAMs) by upregulating the expression of nuclear factor-κB (44). TAMs have emerged as prominent players in brain cancer (24). They are highly plastic cells that integrate input from cytokines, growth factors, and other stimuli, resulting in diverse activation states and cellular phenotypes, including promotion of invasion, angiogenesis, metastasis, and immune suppression (24). HIF could also inhibit DC maturation, inactivate cytotoxic T lymphocytes (CTLs) and target PD-L1 to evade anticancer immune responses (31).

Overall, the immunomodulatory effects of tumor vasculature are important targets in understanding and manipulating the TME. Anti-angiogenic therapy could not only normalize the tumor vasculature, but also transform the immunosuppressive TME to the immunosupportive one to improve anti-tumor immune response.

### ICIs Enhance the Anti-Tumor Effects of Anti-Angiogenic Agents

**Figure 2:**
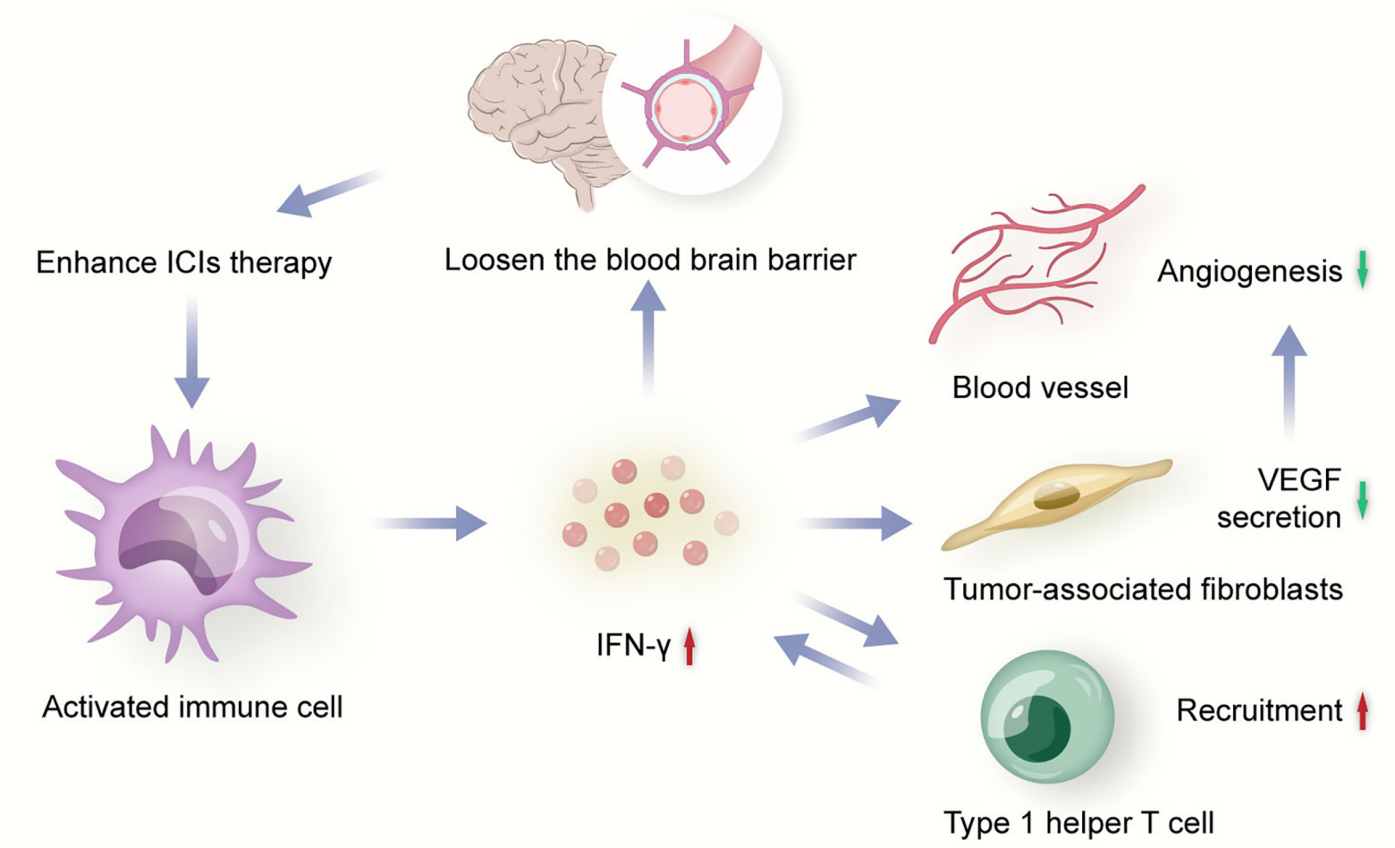
Activated immune cell secrets IFN-γ to inhibit angiogenesis. ICIs, immune checkpoint inhibitors; VEGF, vascular endothelial growth factor.

Tumor immune response is closely influenced by angiogenesis. Meanwhile, tumor angiogenesis also highly depends on immunosuppressive microenvironment. ICIs could activate immune cells to secret immune-mediating cytokine with anti-angiogenesis effects to induce tumor vessel normalization (45). IFN-γ is one of the important mediums during the process (**Figure 2**). For example, the activation of IFN-γ signaling pathway on CD8+T cells might be one of the potential mechanisms of the vasculature-normalizing effect of ICIs (32). IFN-γ could inhibit some pathways inducing angiogenesis, such as Notch signaling pathway, to effectively retard tumor growth (31). IFN-γ could also reduce the VEGF secretion of tumor-associated fibroblasts to down-regulated angiogenesis (31). In addition, IFN-γ could increase expression of CXCL9, CXCL10, and CXCL1 which recruit Th1 cells (46), and Th1 cells could secrete IFN-γ in turn, which is significantly associated with vessel normalization (47). Besides, activated CD4+ T cells in the brain could loosen the BBB to circulating antibodies through local IFN-γ production, which is a mechanism that anti-PD-1/PD-L1 therapy could potentially enhance (48).

Immunosuppressive cells could also stimulate tumor angiogenesis by cooperating with pro-angiogenic factors. For instance, MDSCs could enhance the proliferation and migration of endothelial cells by secreting VEGF, and promote tumor angiogenesis by inducing the production of matrix metalloproteinase 9 to act on the extracellular matrix (31). DC precursors could induce tumor angiogenesis in cooperation with VEGF-VEGFR signaling pathway which could further induce DC precursor endothelial-like specialization and migration to blood vessels (31). Moreover, through the expression and secretion of pro-angiogenic factors, some other myeloid cell subgroups might be also equipped with the ability to promote angiogenesis, including TAMs, Tregs, B cells, monocytes and neutrophils (31). These basic researches have provided the evidence that anti-angiogenic therapy could be more effective following the generation of an immunosupportive microenvironment.

Basic researches have suggested that immune response and angiogenesis are mutually regulated, and alleviated immunosuppression coupled with normalization of the tumor vasculature could achieve a loop of positive feedback that promotes each other (32). Some preclinical studies indicated that the efficacy of ICIs combined with anti-angiogenesis was significantly superior to monotherapy in advanced NSCLC. Sha Zhao et al. demonstrated that based on syngeneic lung cancer mouse model, low-dose apatinib could result in alleviating hypoxia, increasing infiltration of CD8+ T cells, reducing recruitment of TAMs in tumor and decreasing TGF-β level both in tumor and serum (49). They also found that combining low-dose apatinib with anti-PD-L1 antibody could significantly retard tumor growth and metastases, and induce prolonged survival in mouse models (49). Additionally, apatinib could improve the anti-tumor efficacy of anti-PD-l therapy *via* upregulating PD-L1 expression in a syngeneic mouse model (50), which might provide a rationale for this combination strategy in the clinic.

## Clinical Data

Based on the synergistic effect on TME, the combination of ICIs and anti-angiogenic agents has been performed in advanced NSCLC. Although data are still immature, clinical benefits have been obtained from this combination strategy. The results of clinical trials investigating the combination effect of anti-angiogenic agents and ICIs were presented in **Table 1**.

**Table 1:** Clinical trials investigating the combination effect of anti-angiogenic agents and ICIs in NSCLC.

Clinical trial	Phase	Histology	Brain metastases	Treatment	Results	TRAEs
NCT02443324	I	Adenocarcinoma, 21/27 (77.8%) Squamous cell carcinoma, 4/27 (14.8%)	NA	Pembrolizumab plus ramucirumab	ORR, 30% DCR, 85% Median PFS, 9.7 m OS rate at 6 m, 84.9%	Total, 25/27 (92.6%) Grade 3, 5/27 (18.5%) Grade 4-5, 0/27 (0%)
NCT03628521	Ib	Squamous cell carcinoma, 12/22 (54.5%) Adenocarcinoma, 9/22 (40.9%)	4/22 (18.2%)	Sintilimab plus anlotinib	ORR, 72.7% DCR, 100% Median PFS, 15 m	Total, 22/22 (100%) Grade 3, 12/22 (54.5%) Grade 4-5, 1/22 (4.5%)
NCT01454102	I	Non-squamous cell carcinoma	NA	Nivolumab plus bevacizumab	ORR, 8% DCR, 58% Median PFS, 37.1 w OS rate at 1 y, 75%	Total, 11/12 (91.7%) Grade 3, 4/12 (33.3%) Grade 4-5, 0/12 (0%)
IMpower150	III	Non-squamous cell carcinoma	NA	Atezolizumab plus bevacizumab plus carboplatin plus paclitaxel	ORR, 63.5% DCR, 85.3% Median PFS, 8.3 m Median OS, 19.2 m	Total, 371/393 (94.4%) Grade 3-4, 219/393 (55.7%) Grade 5, 11/393 (2.8%)
NCT02039674	I	Non-squamous cell carcinoma	4/25 (16%)	Pembrolizumab plus bevacizumab plus carboplatin plus paclitaxel	ORR, 56% DCR, 76% Median PFS, 7.1 m Median OS, 16.7 m	Total, 23/24 (95.8%) Grade 3-4, 10/24 (41.7%) Grade 5, 0/24 (0%)

NSCLC, non-small cell lung cancer; m, month(s); w, weeks; y, year; NA, not applicable; ICIs, immune checkpoint inhibitors; ORR, objective response rate; DCR, disease control rate; PFS, progression-free survival; OS, overall survival; TRAEs, treatment related adverse events.

Herbst et al. designed a multi-cohort phase I trial (NCT02443324) to assess the effect of ramucirumab plus pembrolizumab in the patients with advanced NSCLC with prior progression on systemic therapy (51). This trial enrolled 27 patients with 77.8% adenocarcinoma and 14.8% squamous cell carcinoma. Median progression-free survival (PFS) was 9.7 months and overall survival (OS) rate at 6 month was 84.9%. Objective response rate (ORR) and disease control rate (DCR) were 30% and 85%, respectively. Treatment related adverse events (TRAEs) occurred in 25 (92.6%) patients with 18.5% grade 3 including adrenal insufficiency, delirium, hypertension, hyponatremia, infusion related reaction, proteinuria and respiratory failure. No grade 4-5 TRAEs occurred. In addition, Chu et al. conducted a phase Ib trial (NCT03628521) to evaluate chemo-free first-line strategy of sintilimab combining anlotinib in treatment-naive and stage IIIB/IV NSCLC patients (52). Twenty-two patients were enrolled in the study and four had baseline brain metastases. The results showed high ORR (72.7%) and DCR (100%) with acceptable tolerability. The incidence rate of grade 3 TRAEs was 54.5%. No grade 4 TRAEs were observed, and one case of grade 5 immune-related pneumonitis occurred. The most common TRAEs were hemorrhage (59.1%), hypothyroidism (50.0%) and hyperuricemia (40.9%). Moreover, Rizvi et al. reported preliminary results from a phase I study (NCT01454102) evaluating the efficacy and safety of nivolumab plus bevacizumab as maintenance therapy in advanced NSCLC without progress on first-line platinum based chemotherapy (53). Median PFS was 37.1 weeks and 1-year OS rate was 75%. TRAEs occurred in 11/12 (91.7%) patients with 33.3% grade 3 and no grade 4 TRAEs. Grade 3 adverse events included pneumonitis, cough and tubulointerstitial nephritis.

IMpower150, a phase III randomized trial, showed a significant prognostic improvement with the addition of atezolizumab to bevacizumab and chemotherapy as first-line treatment for nonsquamous metastatic NSCLC (54). This clinical study enrolled a total of 1202 patients and randomly assigned them to three group, atezolizumab plus bevacizumab plus carboplatin plus paclitaxel (ABCP group, 400 patients), atezolizumab plus carboplatin plus paclitaxel (ACP group, 402 patients) and bevacizumab plus carboplatin plus paclitaxel (BCP group, 400 patients). The results indicated that ABCP group had higher rate of PFS at 12 months and objective response than BCP group, regardless of the PD-L1 expression status. It was worth mentioning that ABCP group also showed significant survival benefit in comparison to BCP group in the patients with sensitizing EGFR mutations and liver metastases (55). However, the frequency of TRAEs did not increase with the addition of atezolizumab and the safety profile was consistent with previously reported safety risks of the individual medicines (54). Although IMpower150 study confirmed successful combination of ICIs and anti-angiogenic agents in metastatic NSCLC, this study excluded patients if they had untreated metastases of the central nervous system. In contrast, a multi-cohort phase I study (NCT02039674) explored the anti-tumor activity and safety of pembrolizumab plus carboplatin-paclitaxel-bevacizumab in advanced non-squamous NSCLC without prior systemic therapy (56). This study randomly assigned patients into 3 cohorts (A, B and C) and the patients in cohort B received pembrolizumab plus carboplatin-paclitaxel-bevacizumab. Cohort B enrolled 25 patients with 4 (16%) brain metastases. ORR was 56% with 1 (4%) complete response and 13 (52%) partial response. Median PFS was 7.1 months and median OS was 16.7 months. TRAEs occurred in 95.8% patients and most events were of mild-to-moderate severity. It should be noted that TRAEs resulted in discontinuation of study treatment in 5 cases in cohort B, including neutropenia, autoimmune colitis, diarrhea, drug hypersensitivity, and pneumonitis.

A real-world retrospective study enrolled 69 patients with NSCLC to explore the efficacy of ICIs combining anti-angiogenesis therapy (57). Sixty-three (91.3%) patients were at stage IV and 16 (23.2%) had sensitizing EGFR mutations. Twenty-nine (42%) patients received nivolumab and 40 (58%) received pembrolizumab. Bevacizumab was used in 45 (65.2%) patients and the remaining patients received apatinib, anlotinib or endostar. ORR was 31.9% and DCR was 89.9%. Median PFS was 8.37 months, while median OS was not reached. It should be noted that the patients receiving combined therapy within 6 months after diagnosis had better ORR than those exceeding 6 months (59.1% *vs*. 19.1%, P = 0.001). These results suggested that it would be better to apply ICIs plus anti-angiogenic agents at the early stage after initial diagnosis. TRAEs appeared in 62% of patients. Most TRAEs were grade 1-2 with only 2 (2.9%) grade 3 (pneumonitis, diarrhea) and no grade 4-5 events. The most common adverse events were fatigue, decreased appetite and nausea.

The combination of ICIs and anti-angiogenic agents showed encouraging anti-tumor activity and tolerable safety profile. Due to the potential neurological sequelae, patients with brain metastases were often excluded from clinical trials. Major ongoing or planned trials investigating ICIs in combination with anti-angiogenic agents in patients with NSCLC (**Table 2**) include NCT03377023 (a trial of nivolumab plus ipilimumab plus nintedanib), NCT03689855 (a trial of atezolizumab plus ramucirumab), NCT03527108 (a trial of nivolumab plus ramucirumab) and NCT02681549 (a trial of pembrolizumab plus bevacizumab) (58). However, at the time of writing, there are no published trial data from prospective randomized controlled trials focusing on the effects of this combination strategy in NSCLC patients with brain metastases which warrant further studies.

**Table 2:** Major ongoing or planned trials investigating ICIs in combination with anti-angiogenic agents in patients with NSCLC.

Clinical trial	Phase	Treatment (arm of combination therapy)	Planned patients	Primary objective
NCT03377023	I/II	Nivolumab plus ipilimumab plus nintedanib	Advanced or metastatic NSCLC	MTD, ORR
NCT03689855	II	Atezolizumab plus ramucirumab	Squamous or non-squamous NSCLC	ORR
NCT03527108	II	Nivolumab plus ramucirumab	Refractory or recurrent advanced NSCLC	DCR
NCT02681549	II	Pembrolizumab plus bevacizumab	Metastatic melanoma or NSCLC	BMRR

ICIs, immune checkpoint inhibitors; NSCLC, non-small cell lung cancer; MTD, maximum tolerated dose; ORR, objective response rate; DCR, disease control rate; BMRR, brain metastasis response rate.

## Predictive Indicators

Despite the promising prospect of immunotherapy and anti-angiogenesis therapy in NSCLC brain metastases, this combination strategy still faces many challenges, one of which is identifying ideal predictive indicators to screen suitable populations. As for anti-angiogenesis therapy, circulating VEGF-A level was evaluated for the prognostic and predictive value in a retrospective analysis (59). This study included five trials involving three types of cancer, AVF2107 (colorectal cancer), E4599 (NSCLC), AVAiL (NSCLC), AVOREN (renal cell carcinoma) and AVF2938 (renal cell carcinoma). In E4599 trial, bevacizumab-based treatment was predictive for PFS benefit in high circulating VEGF-A group (>36 pg/mL) but not in low VEGF-A group. By contrast, circulating VEGF-A level (cutoff value, 45 pg/mL) was not prognostic for PFS and OS in AVAiL trial. Other biomarkers such as VEGFR-2, FGF-2 and IL-8 were proposed and investigated, but none could predict response to anti-angiogenesis therapy (60). Several studies indicated that anti-angiogenic TRAEs and the number of circulating endothelial cells were positively associated with the clinical benefit (60, 61), but none were validated for routine clinical use. Similarly, the use of ICIs for the treatment of intracranial metastatic tumors also requires effective predictive indicators. Previous studies have proven that the expression of PD-L1 and the presence of tumor-infiltrating lymphocytes (TILs) within TME are considered prognostic and predictive markers in patients treated with immunotherapy (62, 63). However, the PD-L1 expression and the presence of TILs might be different in CNS when compared with extracranial sites, with lower PD-L1 expression and less TILs infiltration in brain metastases compared with matched NSCLC primary tumors (64). Tumor mutational burden (TMB) was also a useful biomarker for response to ICIs in advanced NSCLC (65), but its value in brain metastases remains unclear. DNA mismatch repair-deficient (dMMR)/microsatellite instability-high (MSI-H) has also been reported to be able to predict the efficacy of ICIs, but the low frequency in NSCLC limits its clinical application (66).

As for the combination therapy, the phase Ib trial (NCT03628521) indicated that the patients with TMB ≥10 mutations per megabase showed higher ORR than those with TMB <10 mutations per megabase (85.7% *vs*. 63.6%) (52). It is worth mentioning that ORR in the patients with positive and negative PD-L1 expression was 69.2% and 75%, respectively (67). The phase I trial (NCT02039674) showed that patients with PD-L1 TPS ≥50% were seemed to have higher ORR than those with PD-L1 TPS <50% (75% *vs*. 47%) (56). IMpower150 also suggested that high expression of an effector T-cell (Teff) gene signature in the tumor was associated with survival benefit (54). However, in comparison to primary tumor of NSCLC, brain metastasis lesions displayed significant downregulation of genes related to immune response and immune cell activation (68). In addition, it is unclear whether aspects of the tumor vasculature are different in tumors that respond to immunotherapy and those that do not, and if features such as hypoxia or production of pro-angiogenic factors may serve as predictive biomarkers. These problems suggest that it is unlikely to precisely predict the efficacy of immunotherapy and anti-angiogenesis combination in brain metastases through current biomarkers. The specific predictive indicators to distinguish appropriate population need further exploration.

## Discussion

Given recent advances in immunotherapy, emerging clinical evidence suggests that ICIs have anti-tumor effects in brain metastases from NSCLC. The OAK study showed that the hazard ratio (HR) for OS with atezolizumab *vs*. docetaxel was 0.73 for the overall population, 0.74 for patients without brain metastases, and 0.54 for patients with brain metastases (69). Similarly, the KEYNOTE-189 study comparing pembrolizumab plus chemotherapy with chemotherapy alone indicated that the HR for OS was 0.36 for patients with brain metastases, with 0.49 for the overall population and 0.53 for patients without brain metastases (70). A pooled analysis of CheckMate 063, 017 and 057 also demonstrated that nivolumab showed a survival advantage in second-line therapy for stable brain metastases when compared with docetaxel (71). Beyond oncogene-driven NSCLC, ICIs have recently shown promising activity in the CNS in patients with NSCLC brain metastases.

Despite the significant benefits of immunotherapy, there are still some problems such as limited patient response rates and drug resistance. Because of both targeting aspects of the TME, immunotherapy and anti-angiogenesis are expected to mutually enhance the anti-tumor effect through reprogramming the TME from immunosuppressive to immunosupportive, but whether this combination can improve response rate or delay drug resistance of monotherapy remains unclear and needs further clinical studies. Tumors can be categorized as inflamed and non-inflamed phenotypes based on the spatial localization of immune cells with respect to the tumor and stromal compartments (72). Almost all relevant therapeutic advances in the field of immunotherapy have been achieved in inflamed tumors, while non-inflamed tumors tend to respond poorly to ICIs (72). Whether anti-angiogenic therapy could expand the benefits of immune checkpoint inhibition to non-inflamed tumors requires additional researches. Clinically, steroids are frequently used in NSCLC patients with brain metastases with the aim of palliating cancer-related symptoms, but the use of steroids is associated with a lower efficacy of ICIs and a worse outcome (73). A retrospective study suggested that anlotinib could potentially replace glucocorticoids and effectively improve edema from brain metastases but this study only included 13 NSCLC patients with 23 brain metastases (74). Whether anti-angiogenesis can indeed counteract the negative effect of steroids needs further research. Hyperprogression is defined as rapid disease progression during immunotherapy, which is associated with poor survival outcome (75). In theory, rapidly proliferative cancer cells need an abundance of blood supply for nutrition, while bevacizumab could starve these cells of blood supply and nutrients and provide potential benefit (76). However, the clinical data is absent and needs further study.

Although the preliminary clinical results have suggested that immunotherapy and anti-angiogenesis combination could potentially provide significant activity against brain metastases, the field of this combination strategy faces many challenges in the pursuit of overcoming the defect of monotherapy and improving the outcome of patients. Firstly, there are various combination regimens involving ICIs (PD-1, PD-L1 and CTLA-4 inhibitors) and anti-angiogenic agents (anti-VEGF antibody, anti-VEGFR antibody and VEGFR TKIs). Which combination regimen is most effective for brain metastases remains to be answered by more data. Secondly, early phase clinical studies have reported the use time and the dosage of ICIs and anti-angiogenic agents (51–53), and the use of anti-angiogenic agents prior ICIs was seemed to be more beneficial *in vitro* and vivo experiments (77). However, no studies have analyzed the changes of pharmacokinetic and pharmacodynamic profiles of each agent after combinational use. The optimal time and sequence of each agent in the combination are currently unknown. The appropriate dosage of each agent also remains unclear. Thirdly, although preliminary studies have showed acceptable toxicities and tolerance of the combination therapy, those studies are at early phase and the samples are small. The toxicities still require close attention. Finally, lacking of efficient and sensitive predictive indicators for the combination therapy leads to difficult selection of optimal candidates.

In conclusion, although resolving the above problems requires a long distance, the combination of ICIs and anti-angiogenic agents has opened a new door for the treatment of NSCLC patients with brain metastases, and is expected to change the clinical management of those patients in the near future. Further studies are urgently needed to obtain the definitive data for the use of this combination strategy in clinic and facilitate the development of the optimal combination algorithm.

## Author Contributions

LF and DC performed a literature search, interpreted data, and wrote the manuscript. WZ and BY supervised and contributed to the writing process. All authors contributed to the article and approved the submitted version.

## Conflict of Interest

The authors declare that the research was conducted in the absence of any commercial or financial relationships that could be construed as a potential conflict of interest.
